# A Comparative Evaluation of the Periodontal Ligament Cell Viability Using Placentrex, Propolis, and Hanks' Balanced Salt Solution: An In-Vitro Study

**DOI:** 10.7759/cureus.57454

**Published:** 2024-04-02

**Authors:** Krishnamoorthy Shankar Havaldar, Musaffar Thoyalil, Savitha Sathyaprasad, Anshad Zaheer, Dhanya Kamalakshan Belchada, Amith Puthukkulangara

**Affiliations:** 1 Department of Pedodontics and Preventive Dentistry, KVG Dental College and Hospital, Sullia, IND; 2 Department of Pedodontics and Preventive Dentistry, P.S.M. College of Dental Science and Research, Thrissur, IND; 3 Department of Conservative Dentistry and Endodontics, Travancore Dental College, Kollam, IND; 4 Department of Pedodontics and Preventive Dentistry, Mahe Institute of Dental Sciences and Hospital, Mahe, IND; 5 Department of Periodontology, Educare Institute of Dental Sciences, Malappuram, IND

**Keywords:** placentrex, propolis, hbss, storage media, periodontal ligament cell viability

## Abstract

Background and objectives: Most of the dento-alveolar trauma that occurs frequently in childhood is often linked to avulsion injuries. Two considerable elements influencing the prognosis of tooth avulsion after replantation are extraoral dry duration and the characteristics of the storage media to support the viability of cells. The study aimed to compare and assess the effectiveness of Hanks' balanced salt solution (HBSS) (HiMedia Laboratories, Thane, India) and Placentrex (Albert David Limited, Kolkata, India) against propolis (5% and 10%) as storage media in preserving the vitality of periodontal ligament (PDL) cells.

Materials and methods: Four groups with 15 samples each were selected for the study. Sixty recently extracted premolars were left to incubate for 30 minutes in 15-ml falcon tubes containing 2.5 ml of collagenase 0.2 mg/ml in phosphate-buffered saline and 2.4 mg/ml of dispase in HBSS, Placentex, propolis 5%, and propolis 10%. After the addition of bovine serum, it was centrifuged for 4 minutes. Trypan blue 0.4% was utilized to recognize the cells, and a hemocytometer was employed for counting the live PDL cells under a light microscope.

Results: Propolis 5% and 10%, HBSS, and Placentrex all showed statistically significant differences in PDL cell viability; however, HBSS was significantly outperformed by Placentrex and propolis.

Conclusion: Placentrex is a superior substitute storage medium in cases of tooth avulsion as opposed to alternatives such as propolis 5% and 10%.

## Introduction

Orofacial structures and teeth are the main areas affected in the majority of patients subjected to trauma. Despite being often disregarded as a public health concern, childhood injuries represent a substantial risk to children's development and health. Among children aged 0-6 years, maxillofacial injuries account for nearly 18% of the overall physical injuries they incur. The oral cavity is the second-leading site of injury. According to a comprehensive meta-analysis of trauma-related dental injuries, 22.7% of trauma cases worldwide involve primary teeth [1-3].

According to the International Association of Dental Traumatology, one in every two children gets a dental assault, with the most common age range being 8 to 12. The degree of damage might range from minor enamel chipping to serious maxillo-facial trauma. Dentition-related trauma ought to be perpetually treated as a medical emergency and handled quickly and effectively [2]. A physiological solution that strongly resembles the oral setting and helps maintain periodontal ligament cell (PDL) viability after avulsion is known as a medium for storage [3]. The optimal storage medium must possess an osmolarity and pH that are comparable to physiological values. As a consequence of this, no antigen-antibody responses should be observed. It should be conveniently accessible at the site of the accident so that the tooth can be placed safely within it immediately. To reduce the proliferation of bacteria and replacement resorption, it should have anti-bacterial and anti-inflammatory characteristics. Furthering, it should have anti-oxidant qualities to protect the cells from oxygen radical-induced damage from oxidative stress. Extraoral dry duration, along with the medium chosen to store the tooth, affects PDL cell longevity and is regarded as the two important factors affecting an avulsed tooth's prognosis following replantation [4-6].

The PDL cells usually revert to their normal state immediately after a lost tooth is replaced, preferably within five minutes or fewer. However, following more than 15 minutes of dry storage, precursor or stem cells lack their ability to develop into fibroblasts, and when stored for more than 30 minutes, all of the PDL cells remaining on the tooth root have most likely become necrotic [5]. Consequently, the extra-alveolar storage environment has an impact on PDL cells remaining the viability of PDL cells. Prompt reimplantation of an avulsed tooth improves PDL cell survival and speeds up PDL repair in as many as 85% of adult teeth. The psychological conditions of the victim and witnesses, knowledge of the correct strategy of action, the presence of a dentist or dental clinic nearby, and concerns about authorization are other elements that affect the outcome of the replantation process.

When replantation is not practicable right away or within the allotted period, altering the extra-alveolar circumstances by keeping the tooth in a physiological storage medium significantly boosts the PDL cells' survivability rate, which has an advantageous influence on the course of healing. More important than extra-oral time is the transport medium's ability to sustain viable cells to avoid ankylosis and replacement resorption. Several studies have investigated the potential survival of PDL cells in cases of tooth avulsion using various storage media such as saline, milk, tap water, saliva, Hanks' balanced salt solution (HBSS) (HiMedia Laboratories, Thane, India), L-3,4-dihydroxyphenylalanine (L-DOPA), Gatorade, ascorbic acid, propolis, growth factors, cryo-protective agents, catalase supplementation, and green tea extracts [6,7].

The degeneration of PDL fibers in cementum occurs more frequently, and inflammatory root resorption is brought on by the presence of necrotic PDL remnants on the root surface and is the primary cause of the loss of teeth that have been replanted. Avulsed teeth are frequently replanted one to four hours following the avulsion [7]. The aim of this study was to compare the efficacy of HBSS with Placentrex (Albert David Limited, Kolkata, India), 5% propolis, and 10% propolis in preserving viable PDL cells, evaluating their effectiveness in maintaining PDL cell viability.

## Materials and methods

The study comprised 60 premolar teeth that were collected recently but at different time intervals from the late KVG Dental College's Department of Oral and Maxillofacial Surgery, Sullia, India. The study ethical clearance was obtained from the KVG Dental College with institutional review board (IRB) number IEC/KVGDC/20/11/2015.

A total of 60 mandibular or maxillary first or second premolars, recently extracted for orthodontic procedures, with closed root apices and a healthy periodontium, were collected. Teeth with dental caries, periodontal diseases, or fractured crowns or roots were excluded. Every tooth will be left 30 minutes to dry to replicate a clinical study setting. After that, the teeth were collected and split into four groups of fifteen randomly.

Using a curette, the damaged tissues were extracted from the coronal 3 mm of the PDL. The teeth of each test group were air-dried for 30 minutes and then immersed in one of the following storage media for 45 minutes, as follows: Group I: HBSS (stored tooth sample); Group II: Placentrex (stored group); Group III: Propolis 5% (stored tooth specimens); and Group IV: Samples of teeth kept in 10% propolis. The reason for air-drying the teeth for 30 minutes initially is crucial because it helps simulate real-world conditions where avulsed teeth may be exposed to air before proper storage. This period of air-drying mimics the delay that can occur between tooth avulsion and the initiation of appropriate storage procedures. By allowing this interval, the study aims to replicate scenarios encountered in practical situations, providing a more realistic assessment of the effectiveness of the different storage media in preserving PDL cell viability.

After drying and putting each experimental tooth in the storage media, the 2.5 ml collagenase and dispase solutions in phosphate-buffered saline were placed in incubators for half an hour in 15 ml falcon tubes. Following incubation, 50 mL of bovine serum was pipetted into each test tube. Using sterile micropipettes, the supernatant was extracted from each tube after it had been centrifuged for four minutes. The vitality of a cell is evaluated using cells labeled with trypan blue. Because trypan blue exclusion labeling can distinguish between live and non-viable cells, it was used. It is predicated on the idea that living cells' intact cell membranes hinder the absorption of specific dyes. In this procedure, a cell solution is mixed with dye, and the result is a visual examination to see if the cells accept or reject the dye. A hemocytometer operating under a 40x light microscope was used to determine the proportion of live cells. We assessed the dye-absorbing propensity of the cells. The cytoplasm of live cells is translucent, whereas that of non-viable cells is blue. Using the following formulas, the cell counts were completed: total cells minus stained cells x 100/total cells. The viability percentage was calculated as follows: viable cells x 100/total cells. Averaging count per square times dilution factor x 10^4^ was used to obtain the number of cells per milliliter. Cells per milliliter multiplied by the initial fluid volume from which the cell sample was extracted was used to determine the total number of cells.

The data was evaluated using the computer software Statistical Package for Social Sciences (IBM SPSS Statistics for Windows, Version 26.0, Armonk, NY). A significance threshold of p<0.05 was applied. The Shapiro-Wilk test was applied to assess whether the data were normal. The descriptive statistics, the Kruskal-Wallis test, and the Bonferroni post hoc test were used to determine the difference within the group.

## Results

If prompt replantation was not feasible, a variety of transport media were considered to maintain PDL cell viability and prevent root resorption. Transporting the tooth to an environment analogous to the oral cavity was the fundamental idea behind the usage of these media. The presence of viable periodontal cells was the main concern we looked forward to in the tests undertaken. Here, Table 1 shows the viable periodontal cells in four different storage media such as HBSS, Placentrex, and propolis 5% and 10%.

**Table 1:** Comparison of variables SD: Standard deviation, HBSS: Hanks' balanced salt solution p-value was considered significant if <0.05.

Variables	Total cell (log_10_), mean±SD	Viable cell (log_10_), mean±SD	Stained cell (log_10_), mean±SD	Viability, mean±SD
Propolis 10%	5.63±0.27	5.31±0.08	5.35±0.27	50.81±16.38
Placentrex	5.74±0.11	5.57±0.07	5.25±0.01	73.13±7.16
HBSS	5.75±0.57	5.56±0.03	5.31±0.01	69.14±7.56
Propolis 5%	5.68±0.22	5.41±0.16	5.37±0.01	62.95±9.31
p-value (Kruskal-Wallis)	0.74	0.82	0.88	0.0001

The Kruskal-Wallis test reported a statistical insignificance between the groups regarding the total cells, viable cells, and stained cells (P>0.05). This indicated that an almost similar number of viable cell prevalence was observed in both HBSS and Placentrex. A smaller number of viable cells and viability were noticed for the different concentrations of propolis, relative to the other two groups. A significant difference was reported in viability (p=0.0001). The average percentage of periodontal cells preserving the capacity of four groups of storage media where viable PDL cells were maximum in Placentrex, which was almost similar to HBBS, the world standard, and followed by propolis 5% which showed better results than compared to propolis 10%. Table 2 showed multiple comparisons of viable live cells across groups using the Kruskal-Wallis test followed by Bonferroni correction.

**Table 2:** Multiple comparisons of viable cells (live cells)×105 across groups HBSS: Hanks' balanced salt solution p-value was considered significant if <0.05. a (-) negative value for the test statistic suggests that the first group (Sample 1) has a lower mean or median compared to the second group (Sample 2).

Pairwise comparisons of groups					
Sample 1 vs. Sample 2	Test Statistic	Standard Error	Standard Test Statistic	p-Value	Adjusted p-Value
Propolis 10%-Propolis 5%	-6.767	6.218	-1.088	0.276	1.000
Propolis 10%-Placentrex	-19.600	6.218	-3.152	0.002	0.01
Propolis 10%-HBSS	-20.833	6.218	-3.351	0.001	0.005
Propolis 5%-Placentrex	12.833	6.218	2.064	0.039	0.234
Propolis 5%-HBSS	14.067	6.218	2.262	0.024	0.142
Placentrex-HBSS	1.233	6.218	0.198	0.843	1.000
Independent sample Kruskal-Wallis test (n=60)	15.882			0.001	

Statistically significant differences were observed between propolis 10% against Placentrex (p=0.01), and propolis 10% against HBSS (p=0.005). Table 3 shows multiple comparisons of viability percentages across groups using the Kruskal-Wallis test followed by Bonferroni correction.

**Table 3:** Multiple comparisons of viability (%) across groups HBSS: Hanks' balanced salt solution p-value was considered significant if <0.05. a (-) negative value for the test statistic suggests that the first group (Sample 1) has a lower mean or median compared to the second group (Sample 2). **: Highly significant

Pairwise comparisons of groups					
Sample 1 vs. Sample 2	Test Statistic	Standard Error	Standard Test Statistic	p-value	Adjusted p-value
Propolis 10%-Propolis 5%	-9.300	6.321	-1.471	0.141	0.847
Propolis 10%-Placentrex	-19.600	6.321	-3.101	0.002	0.012
Propolis 10%-HBSS	-26.967	6.321	-4.266	0.001	0.001
Propolis 5%-Placentrex	10.300	6.321	1.629	0.103	0.619
Propolis 5%-HBSS	17.667	6.321	2.795	0.005	0.031
Placentrex-HBSS	7.367	6.321	1.165	0.244	1.000
Independent sample Kruskal-Wallis test (n=60)	20.901			0.000**	

Statistically significant differences were observed between propolis 10% against Placentrex (p=0.012), propolis 10% against HBSS (p=0.000), and propolis 5% against HBSS (p=0.031). Independent sample Kruskal-Wallis test showed statistically insignificant difference (p=0.462) across groups for stained non-viable cells. Henceforth, multiple comparisons were not performed.

## Discussion

The vitality of the PDL is critical to the repair of the periodontium and inhibits resorption, which is the primary determinant of the long-lasting success of the reimplanted tooth in avulsion scenarios. There are numerous storage mediums available that help to maintain the vitality of the PDL cells. In this study, an attempt is made to compare and contrast the use of Placentrex, 5%, and 10% of propolis with the golden standard, HBSS solution.

The American Association of Endodontists recommends HBSS as the ideal storage medium for avulsed teeth. It has been found that HBSS helps sustain the vitality of PDL cells for up to 24 hours at 4°C and ambient temperature. The main drawback of HBSS is that they are difficult to find in locations where these traumatic injuries occur. Finding a storage medium that is both practical and widely available is therefore necessary [7]. One potential storage option for avulsed teeth is propolis, which was suggested by many authors. It is comparatively beneficial and possesses a wide range of pharmacological actions, including immune-modulatory, anti-cancer, anti-bacterial, and anti-inflammatory qualities. Additionally, it has been suggested that propolis prevents osteoclastic resorption, a frequent aftereffect of tooth replantation. Certain steps in the process that mature into fully functional osteoclasts are inhibited by propolis [8].

In a study by Payal Saxena et al., they used propolis at various concentrations: HBSS, 0.5% milk, artificial saliva, a combination of various concentrations of propolis, and Dulbecco’s modified Eagle’s medium (DMEM). They found that propolis 10%+DMEM, propolis 20%+DMEM, and DMEM solely work, as well as combinations, as the preferred storage medium to preserve PDL cell viability for up to 24 hours during the extra-alveolar phase. For shorter times, up to 12 hours, other more accessible media, including milk, might be a suitable substitute storage medium. In the present study, we found similar results with the use of propolis at 5% and 10% [9]. Another study by Xiaojing Yuan et al. concluded that the osteogenic differentiation ability and viability of PDL cells were compared to milk, Brazilian propolis, and HBSS. Brazilian propolis, however, has a stronger anti-inflammatory impact [10].

The next medium, Placentrex, is made of fresh human placental extract and nitrogen. It is useful for treating a range of illnesses because of its anti-inflammatory and anti-aging properties. It can aid in the healing of wounds through enhanced blood flow, elevated production of hormones, and quicker tissue repair [11]. De et al. utilized zymography to document the gelatinase/collagenase action of a human placental sample after obtaining a collagenase-active peptide [12]. There are not many studies undertaken with Placentrex being used as a storage medium for avulsion. Because of its properties, it is believed that it can act as a better medium that can preserve the vitality of PDL cells. Few investigations have suggested that Placentrex is a better storage medium for maintaining the vitality of the PDL. Here, we showcased the effect of the usage of four distinct transport media: Placentrex, HBSS, propolis 5%, and propolis 10%. Out of the four media, HBSS showed a significantly superior ability to preserve PDL viability.

The benefits of HBSS include providing an ionic equilibrium, a physiologically regulated solution that is perfect for preserving cells, and the ability to maintain cell viability without promoting cell division. The mean percentage of PDL cells preserving the capacity of four groups of storage media where viable PDL cells are maximum in Placentrex, which is almost similar to HBBS, the world standard, and followed by propolis 5%, which showed better results than compared to propolis 10%.

The study has some limitations such as the study's sample size comprised only four groups with 15 samples each, totaling 60 samples, which may limit the generalizability of the findings and reduce statistical power. Additionally, the study was conducted in vitro using extracted premolars, potentially failing to fully replicate the complex in vivo environment of tooth avulsion injuries. Moreover, the short incubation period of 30 minutes may not accurately reflect real-life dry times experienced in clinical scenarios, and a longer incubation period or variation in dry times could provide more accurate insights. The study did not directly assess clinical outcomes following tooth avulsion and replantation, necessitating future research to correlate in vitro findings with clinical outcomes to guide treatment decisions effectively. Future research addressing these limitations could improve the validity and relevance of the results, allowing for better decision-making regarding the treatment of tooth avulsion injuries.

## Conclusions

The study findings revealed that the HBSS group had notably more viable PDL cells, nearly matching those of the Placentrex sample. PDL cell viability was significantly decreased across both propolis concentrations. Propolis 5% and propolis 10% showed different percentages of viable cells. Comparing Placentrex to propolis 5% and 10%, Placentrex showed more live PDL cells. When contrasted favorably to alternative media, such as propolis 5% and 10%, Placentrex should be considered a feasible option for transporting avulsed teeth. Nevertheless, further research is required to validate the benefits of Placenterex as an appropriate storage medium. In comparison to Placentrex and propolis, HBSS performed significantly better in preserving cell viability.
